# Clinical Validation of Heart Rate Apps: Mixed-Methods Evaluation Study

**DOI:** 10.2196/mhealth.7254

**Published:** 2017-08-25

**Authors:** Thijs Vandenberk, Jelle Stans, Christophe Mortelmans, Ruth Van Haelst, Gertjan Van Schelvergem, Caroline Pelckmans, Christophe JP Smeets, Dorien Lanssens, Hélène De Cannière, Valerie Storms, Inge M Thijs, Bert Vaes, Pieter M Vandervoort

**Affiliations:** ^1^ Mobile Health Unit Faculty of Medicine and Life Sciences Hasselt University Hasselt Belgium; ^2^ Department of Cardiology Ziekenhuis Oost-Limburg Genk Belgium; ^3^ Department of Public Health and Primary Care KU Leuven Leuven Belgium

**Keywords:** heart rate, software validation, remote sensing technology

## Abstract

**Background:**

Photoplethysmography (PPG) is a proven way to measure heart rate (HR). This technology is already available in smartphones, which allows measuring HR only by using the smartphone. Given the widespread availability of smartphones, this creates a scalable way to enable mobile HR monitoring. An essential precondition is that these technologies are as reliable and accurate as the current clinical (gold) standards. At this moment, there is no consensus on a gold standard method for the validation of HR apps. This results in different validation processes that do not always reflect the veracious outcome of comparison.

**Objective:**

The aim of this paper was to investigate and describe the necessary elements in validating and comparing HR apps versus standard technology.

**Methods:**

The FibriCheck (Qompium) app was used in two separate prospective nonrandomized studies. In the first study, the HR of the FibriCheck app was consecutively compared with 2 different Food and Drug Administration (FDA)-cleared HR devices: the Nonin oximeter and the AliveCor Mobile ECG. In the second study, a next step in validation was performed by comparing the beat-to-beat intervals of the FibriCheck app to a synchronized ECG recording.

**Results:**

In the first study, the HR (BPM, beats per minute) of 88 random subjects consecutively measured with the 3 devices showed a correlation coefficient of .834 between FibriCheck and Nonin, .88 between FibriCheck and AliveCor, and .897 between Nonin and AliveCor. A single way analysis of variance (ANOVA; *P*=.61 was executed to test the hypothesis that there were no significant differences between the HRs as measured by the 3 devices. In the second study, 20,298 (ms) R-R intervals (RRI)–peak-to-peak intervals (PPI) from 229 subjects were analyzed. This resulted in a positive correlation (rs=.993, root mean square deviation [RMSE]=23.04 ms, and normalized root mean square error [NRMSE]=0.012) between the PPI from FibriCheck and the RRI from the wearable ECG. There was no significant difference (*P*=.92) between these intervals.

**Conclusions:**

Our findings suggest that the most suitable method for the validation of an HR app is a simultaneous measurement of the HR by the smartphone app and an ECG system, compared on the basis of beat-to-beat analysis. This approach could lead to more correct assessments of the accuracy of HR apps.

## Introduction

**Figure 1:**
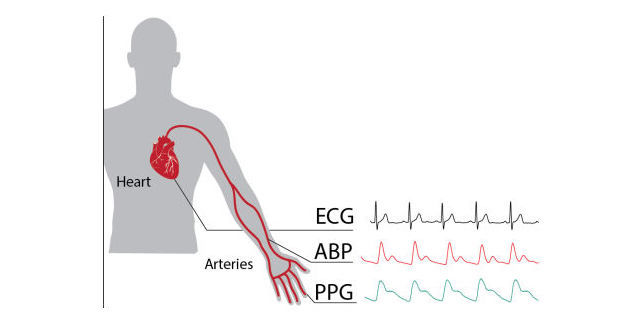
Visualized relation between electrocardiography (ECG), arterial blood pressure (ABP), and photoplethysmography (PPG).

The rapid evolution of technology has brought highly sophisticated electronic devices such as smartphones in our daily lives. The market for these devices is growing at a rapid pace. Globally, there are about 2.6 billion smartphone subscriptions, and by 2020, this number is projected to reach 6.1 billion [1]. Smartphones with multimedia capabilities open new possibilities for app development and service delivery [2]. Recently, smartphones have started to be used for medical purposes to measure numerous vital parameters such as heart rate (HR) and body temperature. This enables the use of a smartphone as a wireless HR monitor [3]. HR is nowadays measured by nurses who have congested schedules and therefore limited time to measure the HR of patients. HR-sensing devices may be a solution for this problem and can be useful in extending the reach of vital signs monitoring in- and outside hospitals, which is typically limited by constraints on human resources [4]. Nowadays, the use of wireless monitors for assessment of HR is a common component of health and fitness programs. Unlike HR apps on smartphones, these HR monitors require a telemetric strap to be worn around the thoracic region or arm to ensure electrocardiography (ECG)-derived HR [5]. The heart is an electromechanical pump with a rhythmic pumping cycle, in which the electrical activity of the heart can be represented in the electrocardiogram by a *P-, QRS-, and T-wave*. For HR and rhythm analysis, the ECG still remains the gold standard. The contraction of the heart propagates a blood pressure pulse wave through the arterial system that travels to the peripheries. A typical arterial blood pressure waveform comprises a systolic upstroke representing the ventricular ejection. After the systolic contraction, the aortic valve closes, which results in a sudden drop in pressure called the dichroic notch [6]. When the pulse pressure wave is passed, these capillaries relax and eject the excessive blood they accumulated, allowing them to return to their initial state. When the areas with dense capillary beds are studied (ie, fingertips, toes, and earlobes), it is possible to observe this pooling of blood by using optical technologies. This technique is also known as the photoplethysmography (PPG) principle. The relationship between ECG, arterial blood pressure (ABP), and PPG is visualized in Figure 1.

PPG is already used in the clinic to measure oxygen saturation and pulse rate [7]. Additionally, it can also be used to estimate cardiac output [8]. PPG used as signal to measure HR is described as the pulse signal. As such, PPG can be used to measure HR without the need for an ECG device. Furthermore, the HR derived from the PPG signal can be used in a series of calculations to determine the heart rate variability (HRV) [9].

PPG is easy to set up, convenient, simple, and economically efficient. It uses a probe that contains a light source and a photodetector to detect the blood volume pulse. The amount of backscattered light corresponds with the variation in blood volume [10]. Hertzman [11] were the first to find a relationship between the intensity of backscattered light and blood volume in 1938.

**Figure 2:**
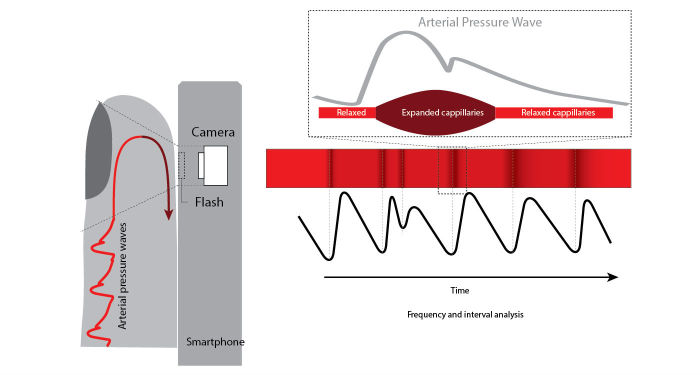
Photoplethysmography (PPG) principle by smartphone.

Traditional PPG systems typically use a narrow wavelength light source (ie, light-emitting diodes [LEDs] with certain colors such as infrared, red, or green) and a specific photodetector to detect PPG signals through the skin. Interestingly, the smartphone camera in combination with the LED flashlight is able to detect these small variations in skin color caused by the blood flow (Figure 2). The camera uses wide-bandwidth pixel-enabling color detection in the red, green, and blue range (RGB-color).

**Figure 3:**
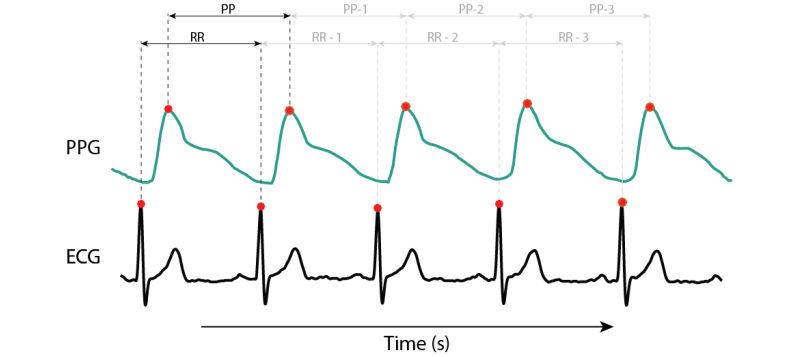
Beat-to-beat analysis from R-R intervals (RRI) and peak-to-peak intervals (PPI).

In 2010, Jonathan and Leahy presented a case study which concluded that HR could indeed be measured through PPG by using a smartphone. This case experiment was confirmed by Gregoski et al in 2011 [5,12]. Currently, numerous smartphone apps exist that measure HR. However, the validity of these apps has not always been confirmed [13]. At this moment, there is no consensus on a gold standard method for the validation of a HR app based on a PPG signal. This results in different validation processes that not always reflect the veracious outcome of comparison. Validation can be done in two ways: (1) by comparing the HR [4,14] or (2) by comparing the ECG- derived R-R intervals (RRI) [15,16] and the PPG-derived peak-to-peak intervals (PPI) [17] as shown in Figure 3. The goal of this paper was to explore which of the two validation approaches is more suited and to investigate and describe the necessary elements in validating and comparing HR apps versus standard technology.

## Methods

To investigate the correct method that should be used to clinically validate smartphone apps that measure HR, the smartphone app FibriCheck was used as test case. For this purpose, two separate, independent, prospective nonrandomized studies were performed. In the first study, the HR as measured by FibriCheck was compared with the HR measured by 2 sequentially used Food and Drug Administration (FDA)-cleared HR-measuring devices. In the second study, the beat-to-beat (RRI/PPI) accuracy of the FibriCheck app was compared with a raw single-lead ECG that was recorded in a synchronized way. For this, a validated and wearable ECG recorder [18] (Imec Holst Centre,) was used. Both studies comply with the Declaration of Helsinki. The study protocol was approved by the local committee on human research, and all participants provided written informed consent.

### Study 1: FibriCheck Compared With FDA-Approved HR Devices

Only 2 FDA-cleared HR measurement devices were used, that is, Nonin oximeter and AliveCor. These 2 devices, which employ different measurement methods, were used to validate a novel smartphone app that measured the participant's HR based on the PPG principle. Nonin uses the transmission PPG method as a stand-alone device, whereas AliveCor uses the ECG as a method measured with a smartphone. The participant's HR was measured 3 times with each measuring device according to the protocol of Terbizan et al [19]. Both FibriCheck and AliveCor were installed on an iPhone 5 (Apple Inc). Participants were recruited in the tertiary care center Ziekenhuis Oost-Limburg (ZOL, Genk, Belgium) in 2015. Inclusion criteria were 18 years or older and able to provide the Dutch written informed consent. Exclusion criteria were failure to obtain valid data with any device or failure to correctly follow the protocol.

**Figure 4:**
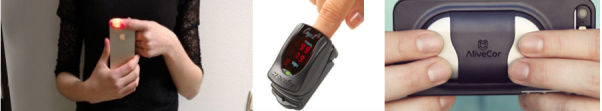
Graphical representation of how measurements are performed using the different devices. Left, FibriCheck application; Middle, Nonin oximeter; Right, AliveCor.

A normalization period of 10 min before the first measurement was used to obtain a resting HR. For standardization, all measurements were performed in the same order, that is, FibriCheck app, Nonin oximeter, and AliveCor. The FibriCheck app measures the HR for 10 s by placing the index finger over the rear camera and LED while holding the smartphone in the other hand (Figure 4, left). Nonin and AliveCor measurements were performed according to the manufacturers’ guidelines.

**Figure 5:**
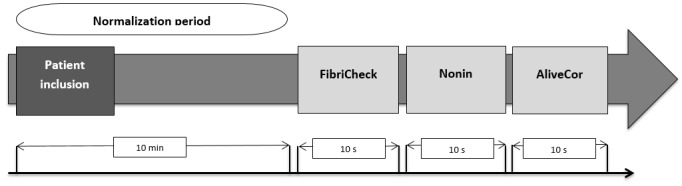
Graphical overview measurement-process study 1.

Figure 5 represents a graphical overview of the step-by-step approach of measurement in study 1. In case of the FibriCheck app, the shown HR result value in beats per minutes (BPM) was used, whereas for both the Nonin oximeter and AliveCor app, the minimum and maximum HR during a 10 s measurement were averaged. Subsequently, all results of HRs measured by the different devices were statistically compared with each other.

The Shapiro-Wilk test was performed to test for normality. Different tests were performed to analyze the results. First, a Pearson correlation test of each possible pair of methods was performed to assess correlation. Second, the agreement between methods was assessed by the construction of Bland-Altman plots of the same pairs. Finally, a paired student *t* test and single-way analysis of variance (ANOVA) test were executed to see whether there was a significant difference between the HR as measured by the different methods. Statistical analysis and generation of Bland-Altman plots were performed by using R statistical software (version 3.2.2).

### Study 2: FibriCheck Beat-to-Beat Accuracy Compared With Wearable ECG in Broad Dynamic Range

**Figure 6:**
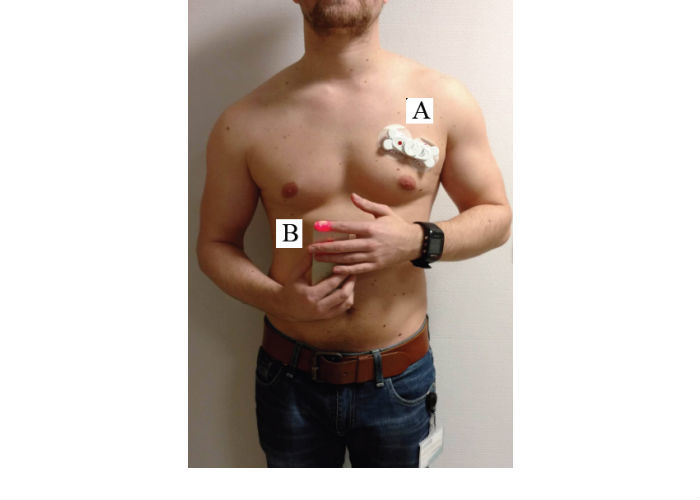
Measurement setup for simultaneous photoplethysmography (PPG) and electrocardiography (ECG) recording. A wearable ECG sensor to measure 1-lead ECG data; B, FibriCheck app to measure PPG data.

The beat-to-beat accuracy of the FibriCheck app was verified by comparing it with a wearable ECG patch. To do so, the FibriCheck smartphone app was used and installed on an iPhone 5S. This app also enables synchronization of the PPG signal, with a simultaneously measured ECG signal of a single-lead wearable ECG patch. This wearable device was attached to the upper left corner of the patient’s chest with 2 disposable electrodes (Figure 6). This enables comparing the raw data of the 2 devices (ie, FibriCheck and wearable ECG) and measurement principles (ie, PPG and ECG). Inclusion criteria were 18 years or older and able to provide the Dutch written informed consent.

Patients with an active pacemaker rhythm were excluded. Patients were either included by a general practitioner (GP) or by a researcher in ZOL between November 2015 and March 2016.

The GP enrolled male and female patients over the age of 65 years, with or without a history or diagnosis of atrial fibrillation (AF). The researcher included subjects who were diagnosed with AF by a 12-lead ECG system and healthy subjects who underwent a sports session. The study population is heterogeneous since it contains patients with a regular or irregular heart rhythm as well as low and high HRs.

Subjects, included by the GP, were measured in a sitting position and asked to perform three consecutive measurements of 60 s. AF patients, included by the researcher, were measured 3 to 6 times in a lying or sitting position. The sports session involved 5 min cycling at a high pace on a stationary exercise bike to reach a maximum HR. Two measurements before and after the exercise were done.

**Figure 7:**
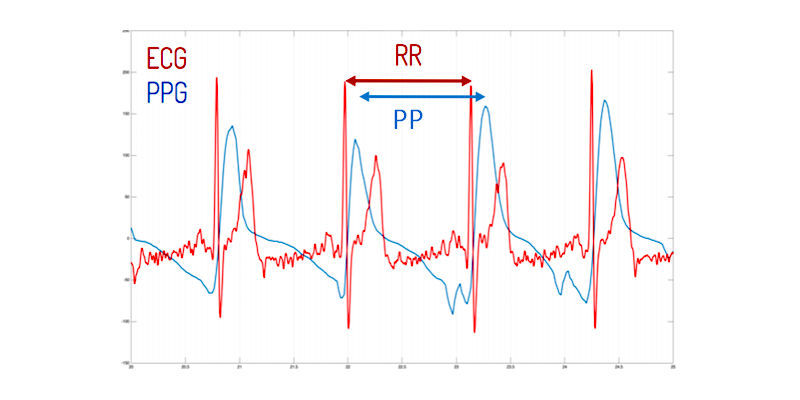
Synchronization of electrocardiography (ECG) and photoplethysmography (PPG) signal.

The FibriCheck app converts 60 Hz video data to raw signals, which were processed with Matlab (Math-Works) to derive the corresponding PPG signal. Time synchronization between ECG and PPG was automatically done by the FibriCheck app. Subsequently, peak detection of the ECG signal and the preconditioned PPG signal was performed by blinded and manual annotation of the identified peaks using Matlab. Finally, it was possible to extract the interpeak distance. An example of the automatic synchronization is shown in Figure 7.

The Kolmogorov-Smirnov test was performed to test for normality. The not normally distributed data are expressed as a median and interquartile range (IQR). A two-sided Wilcoxon signed-rank test was performed to compare two continuous variables for the not-normally distributed data. Correlation between the two continuous variables was calculated by a Spearman correlation test. All analyses were two-sided, and the level of significance was set at a value of .05. Root mean square error (RMSE) and normalized root mean square error (NRMSE) were performed to evaluate the range of errors between predicted and observed values. Data analyses were performed with R statistical software (version 3.2.2). Graphical presentations, such as correlations plots, Bland-Altman plot, and Kernel density plots, were made in RStudio version 0.99.486 (Rstudio Inc).

## Results

### Study 1: FibriCheck Compared With FDA-Approved HR Devices

In total, 91 persons were included in the study. A total of 3 persons were excluded from analysis because of failure to obtain valid data with 1 or more devices. This resulted in a final study population of 88 subjects. Table 1 shows the characteristics of these patients. Data are expressed as mean (standard deviation [SD]).

The HR measurements as acquired by the three different methods were compared for assessing the ability of the FibriCheck app to correctly measure subjects’ HR. First, two-sided Pearson correlation tests were performed to evaluate the correlation between each possible pair of devices. Second, a paired student *t* test was performed. Thereafter, the RMSE and NRMSE were calculated (Table 3).

**Table 1:** Characteristics of patients in study 1.

Variable	Men (n=50)	Women (n=38)	All
Age in years, mean (SD)	49.34 (17.62)	44.63 (17.69)	47.31 (17.70)
Height, mean (SD)	177.14 (8.11)	165.97 (5.22)	172.26 (8.92)
Weight, mean (SD)	82 (14.12)	66.55 (6.56)	75.25 (13.75)

**Table:** 

Measuring device	Heart rate, mean (SD^a^)
Nonin, bpm^b^	69 (12)
FibriCheck, bpm	71 (13)
AliveCor, bpm	69 (12)

^a^SD: standard deviation.

^b^BPM: beats per minute.

**Table 3:** Correlation coefficients, statistical significance, root mean square error, and normalized root mean square error for each pair of devices.

Pair of devices	Correlation coefficient (*r*)	Statistical significance (two-tailed)	Root mean square error (beats per minute)	Normalized root mean square error (beats per minute)
FibriCheck–Nonin	.834	*P*=.36	7.40	0.11
FibriCheck–AliveCor	.88	*P*=.45	6.26	0.09
Nonin–AliveCor	.897	*P*=.87	5.46	0.08

Finally, an ANOVA test was performed to evaluate whether there was a significant difference between the results of the HR measurements of the different devices. The results indicate no significant difference (*P*=.61) between the HRs measured by the 3 different devices.

**Figure 8:**
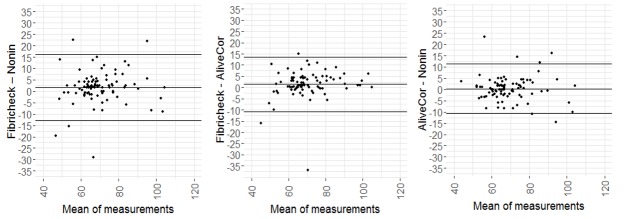
Bland-Altman plots for each device pair. The mean difference (bias), 1.96 (lower limit of agreement, LLA) and +1.96 standard deviations (upper limit of agreement, ULA) are plotted as full lines.

Results show high correlations without significant differences for all device pairs. However, since correlation does not necessarily imply agreement, Bland-Altman plots were constructed to evaluate agreement between each pair of devices (Figure 8).

The mean bias ranged from 0.29 bpm (Nonin–AliveCor) to 1.42 bpm (FibriCheck–AliveCor) and 1.72 bpm (FibriCheck–Nonin). Some measurements were not situated between the lower limit of agreement (LLA) and the upper limit of agreement (ULA).

### Study 2: FibriCheck Beat-to-Beat Accuracy Compared With Wearable ECG in Broad Dynamic Range

A total of 247 subjects were measured with the FibriCheck app in the presence of a GP (n=238) or a researcher in ZOL (n=19). The researcher included both healthy subjects (n=12) and patients who were diagnosed with AF by a 12-lead ECG system (n=7). Around 18 patients from the total study population, all included by the GP, had a pacemaker and were all excluded. Therefore, the final study population included 229 subjects. Table 4 shows the characteristics of these patients.

**Table 4:** Characteristics of patients in study 2.

Variable	Men (n=105)	Women (n=120)	All^a^
Age in years, mean (SD^b^)	73.54 (13.86)	75.51 (14.34)	74.59 (14.12)
Height in centimeters, mean (SD)	173.79 (8.04)	161.22 (7.32)	166.25 (13.79)
Weight in kilograms, mean (SD)	80.3 (13.59)	67.52 (13.83)	73.45 (15.11)
Diabetic, n (%)	20 (19)	24 (20)	44 (20)
Atrial fibrillation, n (%)	50 (47.62)	48 (40)	98 (43.56)
Systolic blood pressure in mm Hg, mean (SD)	128.54 (13.84)	130.6 (20.34)	129.65 (17.13)^c^
Diastolic blood pressure in mm Hg, mean (SD)	74.39 (7.59)	73.55 (11.10)	73.94 (9.62)^c^
CHA_2_ DS_2_-VASc^d^ score, mean (SD)	3.61 (1.75)	4.58 (1.86)	4.13 (1.87)

^a^Demographics of 4 patients were reported as missing data.^b^SD: standard deviation.Systolic and diastolic blood pressure were not included for patients who underwent the sport session.^d^CHA2DS2-VASc calculates the stroke risk for patients with atrial fibrillation.

Table 5 provides the study results. In total, 237 measurements (PPG-ECG pairs) were performed, which resulted in a 20,298 beat-to-beat analysis. An average interval of 758 (RRI) and 758 (PPI) was observed.

**Table 5:** Overview study results.

Variable	ECG^a^	PPG^b^
Number of Intervals	20,298	20,298
Average interval (ms)	758.4 (351.6)	758.2 (333.3)
Minimum value (ms)	312.5	316.7
Maximum value (ms)	2223.0	2233.0

^a^ECG: electrocardiography.

^b^PPG: photoplethysmography.

The Wilcoxon signed-rank test showed no significant difference between ECG and PPG (*P*=.92). To calculate the correlation and difference between the ECG and PPG measurement, the Spearman rank-order correlation, RMSE, and NRMSE were calculated. A correlation of *rs*=.993 was found, with RMSE=23.04 ms and NRMSE=0.012 ms.

**Figure 9:**
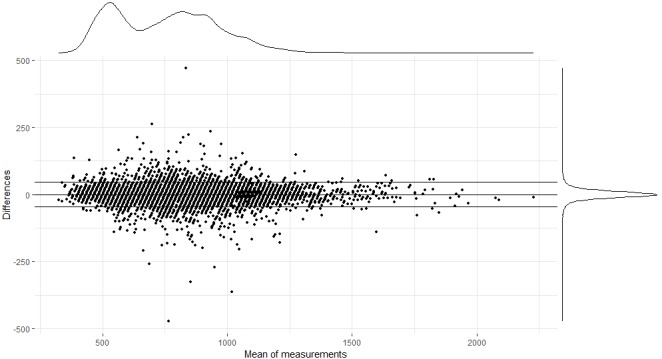
Bland-Altman plot comparing the reference R-R intervals (electrocardiography [ECG]) to the peak-to-peak intervals (photoplethysmography, PPG).

Additionally, a Bland-Altman plot was made, showing the differences between the beat-to-beat intervals of the PPG-ECG pairs in function of the means. The error distribution and the distribution of the mean duration of the intervals of the PPG-ECG pairs are visualized by kernel density plots (Figure 9). The mean bias is 0.26 (23.045) with a 95% CI from −45.82 to 46.35. The CI (μ±1.96σ) is visualized by the dashed lines. An in-depth analysis was performed to investigate differences within the study results. This detailed analysis was based on two categories: low versus high HR and regular versus irregular intervals.

**Figure 10:**
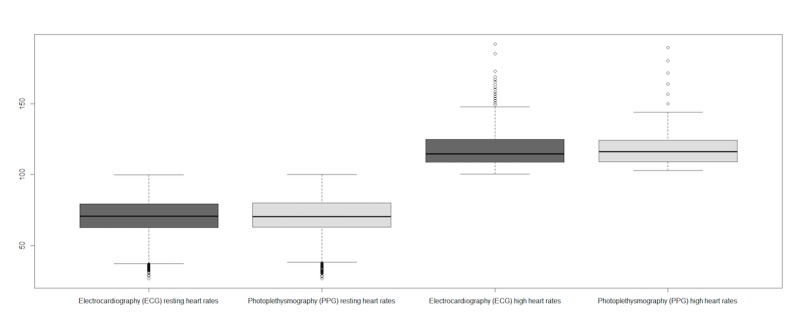
Overview heart rates divided in resting and high heart rates.

On the basis of the definition by Laskowski of a resting HR [20] a distinction was made between a resting HR (40-100 BPM) and high HR (100-170 BPM). Figure 10 visualizes and Table 6 describes the study results for this distinction.

**Table 6:** Summary of intervals divided in low, high, and overall heart rate.

Variable	Interval 40-100 heart rate		Interval 100-170 heart rate	
	ECG^a^	PPG^b^	ECG	PPG
Number of intervals	13,913	13,913	6385	6385
Average interval (ms)	869.5 (203.2)	868.9 (200)	516.4 (70.3)	516.9 (66.6)
Minimum value (ms)	601.6	483.3	312.5	316.7
Maximum value (ms)	2223.0	2233.0	597.7	1000

^a^ECG: electrocardiography.

^b^PPG: photoplethysmography.

**Figure 11:**
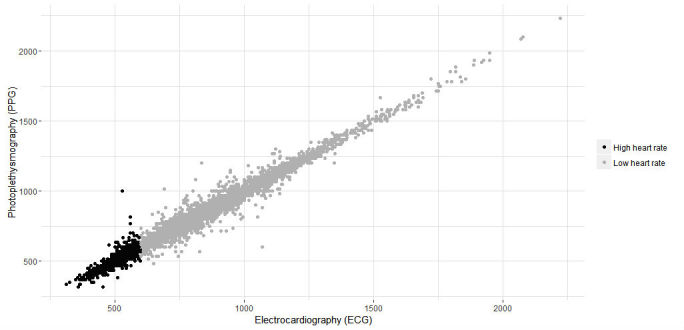
Correlation of intervals for both techniques (ie, electrocardiography [ECG] and photoplethysmography [PPG]) in milliseconds. Gray, high heart rates; Black, low heart rates.

No significant difference was observed between both techniques within the interval 40-100 (*P*=.76) or interval 100-170 (*P*=.69). Correlation of interval 40-100 between ECG and PPG was strong (*rs*=.985; RMSE=25.32 ms and NRMSE=0.014). Interval 100-170 was also strongly correlated (*rs*=.956; RMSE=17.06 ms and NRMSE=0.025). The correlation between both techniques is plotted in Figure 11.

**Figure 12:**
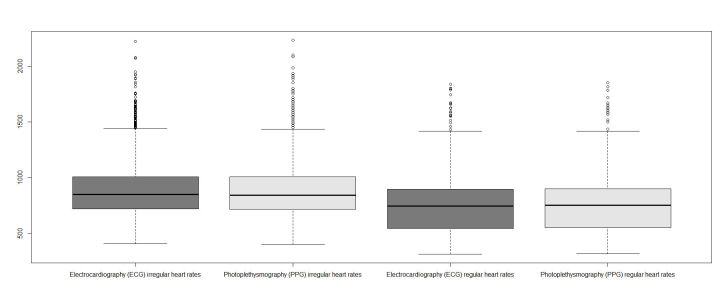
Overview of beat-to-beat intervals divided in irregular and regular beats.

A last step in analysis was performed by investigating the differences between regular and irregular intervals. Figure 12 visualizes the measurements divided into irregular and regular intervals.

Table 7 describes the study results for this category. A total of 2648 intervals were obtained from patients with AF versus 17649 from patients with regular heart rhythms.

**Table 7:** Summary of intervals divided into regular and irregular beat-to-beats.

Variable	Regular		Irregular	
	ECG^a^	PPG^b^	ECG	PPG
Number of intervals	17,649	17,649	2648	2648
Average interval (ms)	738.9 (204.8)	738.6 (205.3)	888.5 (241.4)	888 (241)
Minimum value (ms)	312.5	316.6	406.3	400
Maximum value (ms)	1835.9	1850	2222.6	2233.3

^a^ECG: electrocardiography.

^b^PPG: photoplethysmography.

**Figure 13:**
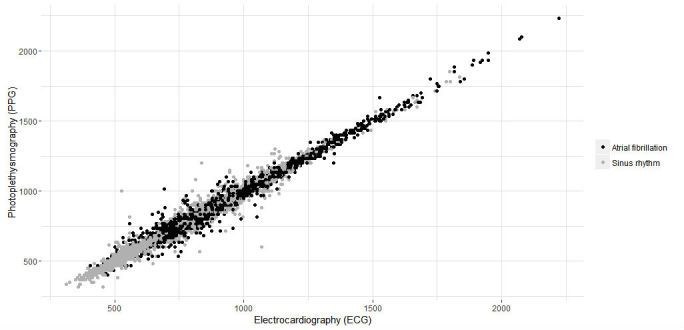
Correlation of intervals for both techniques (ie, electrocardiography [ECG] and photoplethysmography [PPG]) in milliseconds. Gray, irregular intervals; Black, regular intervals.

No significant difference was observed between both techniques within group regular HR (*P*=.92) or group AF (*P*=.93). Correlation for group regular between ECG and PPG was strong (*rs*=.994; RMSE=20.49 ms and NRMSE=0.013). Group irregular was also strongly correlated (*rs*=.9832; RMSE=37.62 ms and NRMSE=0.021). The correlation between both techniques is plotted in Figure 13.

## Discussion

### Principal Findings

We sought to determine an approach to validate an HR-measuring app. For this experiment, we set up two different studies for determining the correct approach to answer the research question. The results were interpreted on the criterion validity (demonstrated by statistical test for a high correlation between new tool and the existing standard) and construct validity (refers to the systematic change in results when the input variable is under varying conditions) as described by Franko [21].

Study 1, FibriCheck compared with FDA approved HR devices, compared 3 tools for measuring HRs in a large sample of volunteers. The tools (Nonin and AliveCor) are approved by the FDA and are already used in clinical practice. The third one is the FibriCheck app. The results of the study, for criterion validity, show a correlation coefficient of .834 between FibriCheck and Nonin, .88 between FibriCheck and AliveCor, and .897 between Nonin and AliveCor. A single way ANOVA, *P*=.61 was executed to construct validity indicating that there is no significant difference between the HRs as measured by the 3 devices.

Study 2, FibriCheck beat-to-beat accuracy compared with wearable ECG, compared the RRI-PPI intervals at the same moment from the FibriCheck app in relation to the data of a wearable ECG. The results of the study show a positive correlation of .993 between RRIs and PPIs. This result supports the validity criteria. For construct validity, no significant difference (*P*=.92) was shown between the intervals from FibriCheck and the intervals from the wearable ECG.

Terbizan et al [19] suggested a minimum correlation of .9 for heart monitors to be clinically reliable. On the basis of the measured results in study 1, no pair of devices complies with this correlation. Terbizan et al suggest to interpret the device as “not reliable.” This is contradictory because both AliveCor and Nonin have an FDA approval. Bland-Altman plots showed some outliers between the devices. In this study, outliers need to be included in the dataset because of the legitimate character of the observation.

A “not reliable” correlation could have multiple causes. For example, there are device-related (eg, different hardware) causes that could influence the signal of the measurement. Furthermore, algorithms converting the PPG signal into HR measurements differ between manufacturers, including in the way they cope with nonperfect measurements. Therefore, when the captured PPG signal is incomplete, for example, because of vibrations or movement by the finger, resulting HR measurements can differ between HR apps and monitors, even when the raw data are identical.

These differing results can be assessed by running the algorithms on a reference database such as the MIT-BIH arrhythmia database for ECG records [22].

In addition, it is important to consider device specifications when evaluating an HR app on the smartphone. The app therefore needs to be validated on a smartphone with minimal device requirements. Smartphones with lower system specifications than required could result in “‘not reliable” results of the app. It is important for the manufacturer of that HR app to ensure the minimal hardware requirements of hardware. This creates the obligation for manufacturers to evaluate apps on multiple smartphones.

Besides possible hardware and algorithm explanations, there could be time-related causes (eg, measurement on different time) that could result in physiological changes causing a change in HR. This could be eliminated by doing synchronous measurements with these devices.

Another explanation could be that taking average of the minimum and maximum HR during a 10-s interval is not the optimal procedure to obtain a reading from these devices. The FibriCheck app gives a single result after a 10-s measurement, whereas the Nonin oximeter gives a continuous reading and the AliveCor a minimum and maximum HR result after 10 s. To address this mismatch, the average of the minimum and maximum HR of a 10-s reading was used in case of the Nonin oximeter and AliveCor.

Further research should be conducted to investigate whether there is a stronger correlation between Nonin and AliveCor than the current results suggest; some suggestions are given below.

Related to the possible time- and analytics-related causes of this result, the next step in validation was performed. Experiment 2 for the beat-to-beat detection between the FibriCheck and an ECG device was set up.

Study 2 shows a positive correlation result of .993, an RMSE of 23.04 ms, and an NRMS of 0.012 for the intervals of the FibriCheck app and ECG device. This means that both methods are almost identical. This result suggests that the FibriCheck app could be used as a clinically validated app for measuring HR. The protocol of study 2 confirms the research question of an approach to validate an HR-measuring smartphone app.

### Study Limitations

Although the results of this study are encouraging, there are a number of limitations to the study that could be taken into account for further research. First of all, the sample comprised both healthy and unhealthy volunteers who were recruited in a hospital setting and in general practice. However, this means that the sample may not be representative of the general population outside the hospital and general practice that could benefit from the smartphone app.

Measurements of the PPG signal could result in multiple limitations. For example, people with small or calloused fingertips may not be suitable for the detection of a PPG signal measured by the smartphone app. They will have inaccurate HR measurements because of problems with light absorption, on which the PPG principle is based. Additionally, patients with poor blood circulation can also show bad signals. Besides physical factors, environmental factors should also be taken into account. For example, ambient temperature has an influence on the blood circulation in the fingertips.

Study 1 coped with some specific limitations based on the study protocol. Due to the need to use both hands for the AliveCor and FibriCheck app, HRs from the 3 different devices were measured sequentially, leading to time intervals of about 30 s to 1 min between the different measurements. This could cause small changes in HR because of small physiological changes. Another limitation of nonsimultaneous HR measurement could be a learning effect for using a mobile HR app. This learning effect could result in a (small) decrease in HR.

There were a number of measurements that fell outside the LLA and ULA. These deviations could be caused by several factors compromising an optimal reading. For example, it could be possible that pressing too hard on the smartphone’s camera impairs the possibility of a good PPG measurement.

For the Nonin oximeter, an incorrect positioning of the device on the finger could hamper a correct reading. Further research could assess whether incorrect usage of these devices can cause deviant HR readings and how to optimally instruct people to avoid these errors.

### Conclusions

Smartphones with multimedia capabilities open new possibilities for app development and service delivery [2]. In the last decades, smartphone apps that measure different vital parameters, such as HR, were developed. At this time, apps that measure the HR of a subject can be installed on a variety of smartphones [10]. However, the validity of these apps has not always been confirmed. This paper describes an approach for the clinical validation of an HR app. The current findings suggest that the most suitable method for the validation of an HR app is a simultaneous measurement of the HR by the smartphone app and an ECG system and comparing the obtained intervals. This approach could lead to almost exact accuracy in the clinical setting. Further studies are needed to evaluate the accuracy outside the hospital and in daily life of subjects.

## Abbreviations

ABP: arterial blood pressure
AF: atrial fibrillation
ANOVA: analysis of variance
BPM: beats per minute
ECG: electrocardiography
FDA: Food and Drug Administration
GP: general practitioner
HR: heart rate
HRV: heart rate variability
IQR: interquartile range
LED: light-emitting diode
LLA: lower limit of agreement
NRMSE: normalized root mean square error
PPI: peak-to-peak interval
PPG: photoplethysmography
RGB: red, green, and blue
RMSE: root mean square error
RRI: R-R intervals
SD: standard deviation
ULA: upper limit of agreement
