# Metadata Correction: Clinical Validation of Heart Rate Apps: Mixed-Methods Evaluation Study

**DOI:** 10.2196/mhealth.9509

**Published:** 2018-03-14

**Authors:** Thijs Vandenberk, Jelle Stans, Christophe Mortelmans, Ruth Van Haelst, Gertjan Van Schelvergem, Caroline Pelckmans, Christophe JP Smeets, Dorien Lanssens, Hélène De Cannière, Valerie Storms, Inge M Thijs, Bert Vaes, Pieter M Vandervoort

**Affiliations:** ^1^ Mobile Health Unit Facultiy of Medicine and Life Sciences Hasselt University Hasselt Belgium; ^2^ Department of Cardiology Ziekenhuis Oost-Limburg Genk Belgium; ^3^ Department of Public Health and Primary Care KU Leuven Leuven Belgium

The authors of “Clinical Validation of Heart Rate Apps: Mixed-Methods Evaluation Study” (JMIR Mhealth Uhealth 2017;5(8):e129) overlooked crediting Christophe Mortelmans, Ruth Van Haelst, and Bert Vaes as authors when metadata was entered into the submission system. They are researchers (described in the paper as general practitioners) with the Department of Public Health and Primary Care, KU Leuven, Leuven, Belgium. Their contributions to this paper were significant, and the authors apologize for the omission in the original article.

The corrected article will appear in the online version of the paper on the JMIR website on March 14, 2018, together with the publication of this correction notice. Because this was made after submission to PubMed or Pubmed Central and other full-text repositories, the corrected article also has been re-submitted to those repositories.

